# Tailoring Mechanical and Soft Magnetic Properties in (Fe_7_Co_6_Ni_6_)_93-x_Ta_x_Al_7_ Multi-Principal Element Alloys: The Role of Ta Addition

**DOI:** 10.3390/ma19122509

**Published:** 2026-06-10

**Authors:** Shizhan Zhang, Wei Wang, Mingyang Li, Zhaoyang Cheng, Jing Liu, Yao Qiu

**Affiliations:** 1 State Key Laboratory of Advanced Refractories, Wuhan University of Science and Technology, Wuhan 430081, China; 19948079392@163.com (S.Z.); 19855513031@163.com (W.W.); limingyang076@gmail.com (M.L.); chengzhaoyang@wust.edu.cn (Z.C.); 2 Guangdong Provincial Key Laboratory of New Energy Materials Service Safety, College of Materials Science and Engineering, Shenzhen University, Shenzhen 518060, China; liujing33@szu.edu.cn; 3 Faculty of Materials, Wuhan University of Science and Technology, Wuhan 430081, China; 4 Faculty of Science, Engineering, and Built Environment, Deakin University, Waurn Ponds, VIC 3216, Australia

**Keywords:** multi-principal element alloys, mechanical properties, soft magnetic property

## Abstract

The growing demand for high-strength and low-core-loss soft magnetic materials in high-efficiency energy conversion devices necessitates the development of novel alloys that combine excellent mechanical and soft magnetic properties. This work investigated the effect of Ta content on the microstructure and properties of as-cast (Fe_7_Co_6_Ni_6_)_93-x_Ta_x_Al_7_ (x = 3, 5, 7) multiprincipal element alloys (MPEAs). Microstructural characterization and mechanical and magnetic testing were conducted using scanning transmission electron microscopy (STEM), tensile testing, and vibrating sample magnetometry (VSM). The alloys featured an FCC matrix, in which Ta addition led to the precipitation of a Ta-rich Laves phase and significant grain refinement. The Ta5 alloy demonstrated an optimal balance of properties, with a yield strength approaching 992 MPa, an elongation of 10%, a saturation magnetization (M_s_) of 94.16 emu/g, and a coercivity of 6.69 Oe, indicating a good balance of strength, ductility, and soft magnetic performance. An appropriate amount of Ta enhanced strength via precipitation and grain-boundary strengthening, while the M_s_ showed only a moderate reduction.

## 1. Introduction

Soft magnetic materials (SMMs) are characterized by their ease of magnetization and demagnetization, typically exhibiting high saturation magnetic flux density and low coercivity (below 1000 A/m). They play a vital role in new energy vehicle motors, air conditioning motors, and transformers [1,2,3]. Current research on SMMs primarily focuses on silicon steel [4,5,6,7], Fe-Ni alloys [8,9], Fe-Co alloys [10], and amorphous metallic alloys [11]. In recent years, the growing demand for SMMs under high-load conditions and the trend toward high-speed motors with compact size, high efficiency, and enhanced durability have driven research on high-strength electrical steels [12,13,14]. The development of such high-strength electrical steels often involved minor additions of other alloying elements to iron-silicon alloys. For instance, recent studies on Cu-containing high-strength electrical steels have reported a yield strength of approximately 900 MPa, but elongation was reduced to 5–8%, indicating relatively poor ductility [4,5]. Furthermore, although some amorphous soft magnetic materials exhibited high yield strength, they were typically limited to thin ribbons, wires, or powders due to the high cooling rates required for their synthesis and often suffered from high brittleness [15,16]. Therefore, designing novel alloys that combine excellent mechanical and soft magnetic properties remains an important research direction.

High entropy alloys (HEAs), also referred to as compositionally complex alloys (CCAs) [17] or multiprincipal element alloys (MPEAs) [18], are a novel class of metallic materials composed of multiple principal elements in near-equiatomic proportions. Since the concept was proposed twenty years ago [19,20], their unique four core effects [21,22] and vast compositional design space have enabled MPEAs to exhibit exceptional properties rarely achieved in conventional alloys, such as outstanding mechanical strength, fracture toughness, and corrosion resistance [23,24,25,26,27]. Based on their constitutional elements, MPEAs can be classified into distinct types, each associated with promising applications. Among the earliest systems studied were those containing 3d transition elements such as Cr, Mn, Fe, Co, and Ni. These alloys featured highly tailorable microstructures and mechanical properties [28]. It was also shown that lightweight MPEAs composed of light elements such as Al, Ti, and V exhibited high specific strength, showing great potential for weight-sensitive applications, including aerospace [17,29,30]. Refractory MPEAs based on W, Ta, Mo, and other refractory elements are suitable for extremely high-temperature environments due to their excellent high-temperature strength and thermal stability [31,32,33].

As research on MPEAs has deepened, investigators have moved beyond an exclusive focus on their mechanical properties. While pursuing breakthroughs in balancing alloy strength and ductility, extensive studies have also examined their corrosion and radiation resistance [34,35] and their physical properties (e.g., hydrogen storage, electrocatalysis, and magnetic properties) [36,37]. Scholars began investigating the magnetic properties of MPEAs around 2010 [38,39]. A series of MPEAs composed of FeCoCrNi combined with Al, Cu, or Pb exhibited M_s_ (<41 emu/g) significantly lower than that of pure Fe (218 emu/g). However, the coercivity of CoCrFeNiCuAl MPEA was measured at only 45 Oe, indicating its potential as a soft magnetic material and thereby initiating research into soft magnetic MPEAs.

Currently, the most common phases in soft magnetic MPEAs are FCC and BCC. Studies on FeCoNiAl MPEAs [40,41,42,43] demonstrated that with increasing Al content, the phase constitution of FeCoNi alloys transitioned progressively from a single-phase FCC structure to dual-phase FCC + BCC, and finally to a single-phase BCC structure. This phase transformation consequently led to alterations in both magnetic and mechanical properties. Regarding magnetic performance, although studies on dual-phase soft magnetic MPEAs showed that the FCC phase exhibited higher M_s_ than the BCC phase [44], the phase structure did not directly determine the M_s_. Instead, it was passively influenced by the differences in ferromagnetic element content. Regarding coercivity, a more complex phase structure and a higher volume fraction of secondary phases generally led to increased coercivity [41]. Regarding mechanical properties, the FCC phase in soft magnetic MPEAs typically exhibited superior ductility but lower strength. In contrast, the BCC phase generally possessed higher strength but greater brittleness. Therefore, the design of high-strength soft magnetic MPEAs should adhere to the following strategy: a ferromagnetic FCC matrix (comprising Fe, Co, Ni) ensures high M_s_, while a minor addition of non-ferromagnetic elements introduces secondary phases for strengthening. This approach enhances mechanical properties via precipitation strengthening, provided that the coercivity remains within the soft magnetic limit (e.g., <1000 A/m), thereby minimizing the detrimental impact on magnetic performance.

Building on the FeCoNiAl base system, existing research has systematically expanded the compositional space by incorporating additional alloying elements. This has led to the development of several notable soft magnetic MPEAs, such as FeCoNiAlSi [44,45,46], FeCoNiAlTa [47,48,49,50], FeCoNiAlMn [51,52,53], and FeCoNiAlCr [54,55,56]. Zhang et al. [44] studied the FeCoNiAlSi soft magnetic MPEA; the as-cast FeCoNi(AlSi)_0.2_ alloy achieved an M_s_ of 1.15 T, a coercivity of 1400 A/m, a compressive yield strength of 342 MPa, and a fracture strain of 50%. Although the coercivity slightly exceeded the limit for soft magnetic materials, it still demonstrated potential as a soft magnetic MPEA. Recently, Lu et al. [41] fabricated an FeCoNiAlMn MPEA via cold-rolling (CR) and annealing. The alloy annealed at 850 °C exhibited superior properties compared with other annealing conditions, achieving a yield strength and elongation of approximately 1 GPa and 24%, respectively, with an M_s_ and coercivity of 120 emu/g and 455 A/m, respectively. Rao et al. [43,47,48,49] conducted a series of studies on FeCoNiAlTa soft magnetic MPEAs. By hot rolling, they obtained fine nanoscale Ni- and Ta-rich L1_2_ precipitates within the FCC matrix, yielding high-strength soft magnetic MPEAs. While maintaining an M_s_ of 88 emu/g, the alloy achieved a yield strength of 1202 MPa, an ultimate tensile strength of 1526 MPa, and an elongation of 15.3%. Concurrently, the role of Al in the FeCoNiAlTa system was investigated, revealing that Al addition enhanced the strength of MPEAs while reducing the M_s_. In subsequent research [48], an FeCoNiTaAl alloy obtained by hot rolling followed by appropriate quenching exhibited a yield strength of 904 ± 11 MPa and an ultimate tensile strength (UTS) of 1336 ± 21 MPa, with an elongation of 53.6 ± 1.5%. It also exhibited an extremely low coercivity of 78 A/m and a moderate M_s_ of 100 emu/g. In the most recent study [49], the hot-rolled and homogenized non-equiatomic Fe_35_Co_30_Ni_30_Ta_5_ alloy was further processed by cold rolling, achieving a yield strength of nearly 2 GPa while maintaining an elongation of 12.6% and an M_s_ of 115.4 Am^2^·kg^−1^. The FeCoNiTaAl soft-magnetic MPEAs have demonstrated excellent balanced properties but still have considerable research potential. Existing studies have primarily focused on the effects of Al content, rolling, and heat treatment on microstructure and properties. In contrast, systematic investigations of variations in the key precipitate-forming element, Ta, remain scarce. In addition, current high-performance soft magnetic MPEAs typically rely on complex thermo-mechanical processing. Consequently, the phase constitution and microstructure of the as-cast FeCoNiTaAl system remain poorly characterized, which hinders the optimization of its composition and production processes.

The optimal Ta content in the FeCoNiTaAl system remains unclear. To elucidate the influence of Ta content on the microstructure, magnetic properties, and mechanical performance of MPEAs and thereby provide critical guidance for subsequent alloy design, this work prepared as-cast (Fe_a_Co_b_Ni_b_)_93-x_Ta_x_Al_7_ (x = 3, 5, 7; a:b = 7:6) alloys via arc melting. This approach aims to introduce an appropriate amount of secondary phase into the FCC matrix by adjusting the Ta content, while simultaneously controlling the quantity and size of these phases to enhance mechanical properties without causing a sharp increase in coercivity. It is expected to achieve a favorable balance between soft magnetic properties and mechanical performance while simplifying alloy fabrication. Furthermore, using microstructural characterization methods such as XRD, SEM, EPMA, and TEM, as well as vibrating sample magnetometry and tensile testing, this work investigated variations in mechanical and soft magnetic properties and the underlying microstructural causes in the FeCoNiAlTa system, thereby guiding adjustments to alloy composition and heat-treatment processes. This work systematically investigates the FeCoNiAlTa system using a combination of microstructural characterization (XRD, SEM, EPMA, TEM) and property measurements (vibrating sample magnetometry (VSM), tensile testing). The aim is to correlate the evolution of mechanical and soft magnetic properties with their underlying microstructural causes, thereby providing insights for optimizing alloy composition and heat treatment.

## 2. Materials and Methods

### 2.1. Alloy Preparation

The (FeCoNi)_93-x_Ta_x_Al_7_ (x = 3, 5, 7; Fe:Co:Ni = 7:6:6) MPEAs were synthesized by arc-melting using high-purity (>99.99 wt.%) constituent elements. With a fixed Al content of 7 at.%, the Ta addition was balanced by proportionally reducing the Fe, Co, and Ni content to maintain the 7:6:6 ratio (see Table 1). The resulting alloys, designated as Ta3, Ta5, and Ta7, were suction-cast into as-cast plates measuring 110 × 30 × 5 mm^3^ for subsequent analysis.

### 2.2. Microstructural Characterization

XRD analysis was performed using a Smart Lab SE X-ray diffractometer (Rigaku, Tokyo, Japan) with a Cu-K_α_ radiation source, with a scanning speed of 2°/min over a 2θ range of 20–100° and operating parameters set at 40 mA and 40 kV. For electron probe microanalysis (EPMA), specimens with dimensions of 5 × 5 × 5 mm^3^ were prepared by first removing the surface oxide layer with 400-grit SiC paper, subsequently grinding with progressively finer sandpaper up to 5000-grit, and finally polishing with a 0.05 μm SiO_2_ suspension. Backscattered electron (BSE) imaging and energy-dispersive X-ray spectroscopy (EDS) analysis were performed using a Shimadzu EPMA-8050G field emission electron probe microanalyzer (Shimadzu, Kyoto, Japan). EDS mapping and point analyses were performed at an accelerating voltage of 15 kV and a current of 100 nA. For EBSD specimens, after initial mechanical polishing, they underwent a two-step finishing procedure. First, they were vibration polished with a 0.05 μm SiO_2_ suspension using a VibroMet™ 2 polisher (Buehler, Chicago, IL, USA). Subsequently, the surface quality was further enhanced by Ar-ion milling using a Gatan PECS II 685 system (Gatan, Pleasanton, CA, USA), with ion-beam energies ranging from 0.1 to 8 keV. Electron backscatter diffraction (EBSD) analysis was performed to determine grain size and to examine post-tensile fracture morphology. The analysis was conducted on an Apreo S Hivac field-emission scanning electron microscope (SEM) (Thermo Fisher Scientific, Waltham, MA, USA) equipped with an EBSD system, using a step size of 1.5 μm. The acquired data were processed using AZtecCrystal 2.1, in which grain sizes were quantified using the equivalent circle diameter method.

TEM specimens were prepared as 3 mm disks, which were mechanically ground to approximately 70 μm in thickness using 2000–5000 grit sandpaper. Thin regions were subsequently created using a Struers TenuPol-5 twin-jet electropolishing system with an electrolyte consisting of 10 vol.% perchloric acid and 90 vol.% methanol. Liquid nitrogen was used to maintain the electrolyte temperature at −20 °C during electropolishing. Microstructural characterization of the MPEAs was performed using a JEM-F200 (CF-HR) scanning transmission electron microscopy (STEM) (JEOL, Akishima, Japan). The microscope was operated at 200 kV, providing a lattice resolution of 0.1 nm for high-resolution TEM (HRTEM) imaging and a probe size below 2 nm for nanoscale compositional mapping via STEM EDS. Observations focused on STEM-EDS analysis and high-resolution imaging of the matrix and secondary phases, including selected-area electron diffraction (SAED) for phase identification.

### 2.3. Soft Magnetic Properties

The room-temperature magnetic properties were measured using a Lake Shore Model 8604 vibrating sample magnetometer (VSM) (Lake Shore Cryotronics, Westerville, OH, USA). For each alloy, three specimens (2 mm^3^) were prepared by grinding with 400-grit sandpaper to remove surface oxides. The magnetic field was swept from −10,000 Oe to +10,000 Oe. Coercivity was determined with high accuracy using a fine step size of 10 Oe near the zero-field region. The saturation magnetization (M_s_) and coercivity (H_c_) reported in the results were average values with standard deviations.

### 2.4. Mechanical Properties

Tensile tests were performed on an Instron Model 3382 testing machine (Instron, Norwood, MA, USA) at a strain rate of 1 × 10^−3^ s^−1^. Dog-bone-shaped specimens (gauge dimensions: 12 mm × 6 mm × 1.3 mm) were used. For each alloy condition, at least 3 specimens were tested to ensure reproducibility.

Vickers hardness was measured using an HV-1000A microhardness tester (Anyote, Shanghai, China) under a load of 500 g, and a dwell time of 10 s. At least ten indentations were made on well-polished surfaces of each alloy, and the average hardness value, along with the standard deviation, was presented.

## 3. Results and Discussion

### 3.1. Microstructure of MPEAs

**Figure 1:**
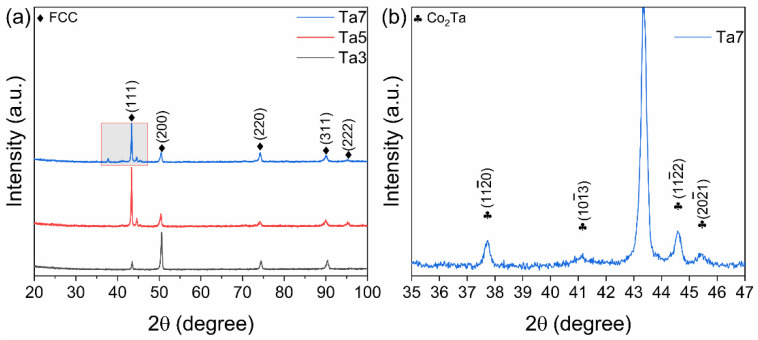
(**a**) XRD patterns of (Fe_7_Co_6_Ni_6_)_93-x_Ta_x_Al_7_ (x = 3, 5, 7) MPEAs; (**b**) XRD pattern of the (Fe_7_Co_6_Ni_6_)_86_Ta_7_Al_7_ alloy in the 2θ range from 35 to 47°.

Figure 1a shows the XRD patterns of the (Fe_7_Co_6_Ni_6_)_93-x_Ta_x_Al_7_ (x = 3, 5, 7) alloys. The presence of diffraction peaks from crystallographic planes such as (111), (200), and (220) indicated that the matrix of these MPEAs possessed a single-phase FCC. The lattice parameters of the FCC phase in these three alloys, calculated from the XRD results, were 3.6026 Å, 3.6134 Å, and 3.6120 Å, respectively. Figure 1b displays a magnified view of the XRD pattern for the Ta7 alloy, revealing minor diffraction peaks from a secondary phase. This phase was identified as a C14 Laves phase with a Co_2_Ta-type structure (PDF#15-0039), adopting the MgZn_2_ prototype and commonly forming in as-cast Ta-containing alloys [57,58,59]. These same diffraction peaks were less pronounced in the Ta5 alloy, being clearly observable only for the ($11\overset{-}{2}2$) plane. No distinct secondary phase diffraction peaks were detected in the XRD pattern of the Ta3 alloy.

**Figure 2:**
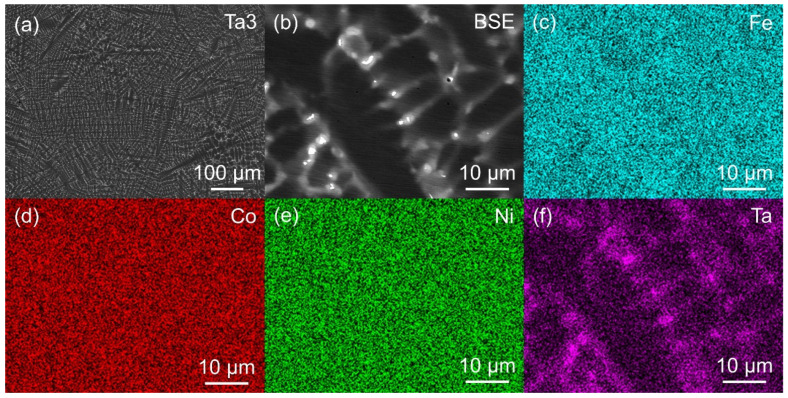
(**a**,**b**) BSE images of the (Fe_7_Co_6_Ni_6_)_90_Ta_3_Al_7_ alloy at lower and higher magnifications; (**c**–**f**) EDS mapping results corresponding to the region in (**b**).

Figure 2 shows BSE images at different magnifications and EDS elemental mapping results of the Ta3 alloy. The low-magnification BSE image in Figure 2a revealed a typical as-cast microstructure of the Ta3 alloy. By combining higher-magnification BSE images with corresponding EDS maps of Fe, Co, Ni, and Ta, it can be seen that the brighter and gray regions corresponded to Ta-rich areas, while the darker regions were Ta-depleted. Al element was not included in Figure 2 due to its low concentration, which prevented meaningful information from being obtained. Co and Ni were uniformly distributed in the alloy, while the Fe element showed some enrichment in the darker regions. Still, the degree of enrichment was relatively weak, thus requiring further TEM-EDS analysis for verification.

**Figure 3:**
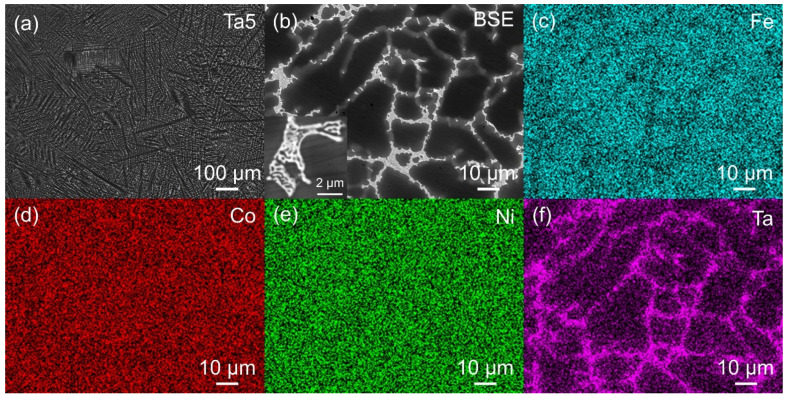
(**a**,**b**) BSE images of the (Fe_7_Co_6_Ni_6_)_88_Ta_5_Al_7_ alloy at lower and higher magnifications; (**c**–**f**) EDS mapping results corresponding to the region in (**b**).

Figure 3 shows the BSE image and elemental mapping results of the Ta5 alloy. The area fraction of the brighter Ta-rich regions was increased. Although the low-magnification BSE image in Figure 3a exhibited a morphology similar to that of Ta3, the higher magnification BSE image revealed a microstructure distinct from Ta3. Finer structural features were observed within the Ta-rich regions of Ta5, as shown in the inset at the lower-left corner of Figure 3b, where alternating brighter and darker fine-scale structures were visible within the Ta-rich regions.

**Figure 4:**
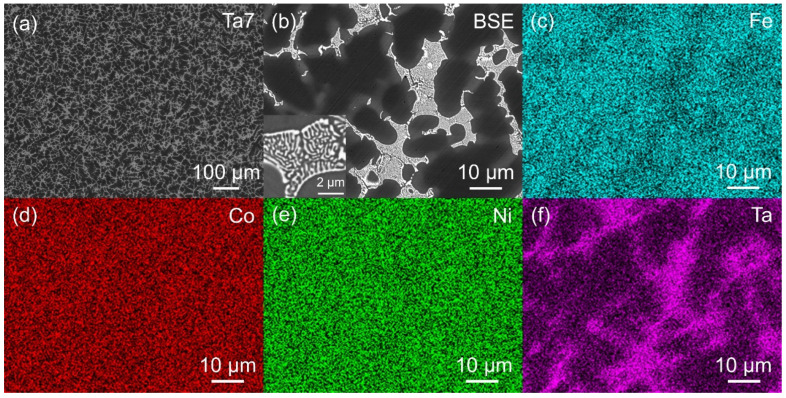
(**a**,**b**) BSE images of the (Fe_7_Co_6_Ni_6_)_86_Ta_7_Al_7_ alloy at lower and higher magnifications; (**c**–**f**) EDS elemental mapping results corresponding to the region in (**b**).

As observed in Figure 4a,b, the Ta7 alloy exhibited a further increase in the fraction of Ta-rich regions, while a reduced fraction of matrix. Furthermore, the fine lamellar structures within the bright Ta-rich regions in Figure 4b were more distinct than those in Ta5, as clearly visible in the inset at the lower-left corner of Figure 4b. Additionally, the Fe element (Figure 4c) showed noticeable enrichment in the matrix. However, the detailed lamellar structures within the Ta-rich regions and the elemental distribution in these regions require further higher-resolution TEM characterization and EDS analysis.

**Figure 5:**
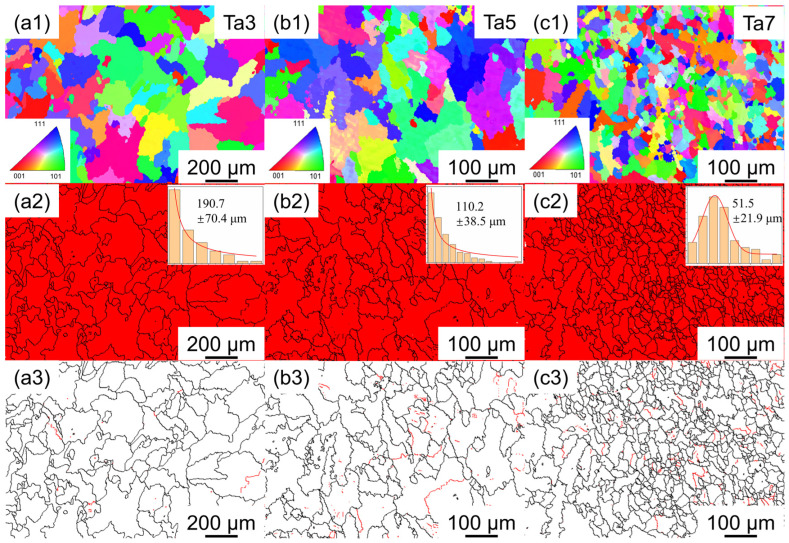
(**a1**–**c1**) Inverse pole figure maps of (Fe_7_Co_6_Ni_6_)_93-x_Ta_x_Al_7_ (x = 3, 5, 7) MPEAs; (**a2**–**c2**) phase maps with grain size distribution histograms; (**a3**–**c3**) grain boundary maps of (Fe_7_Co_6_Ni_6_)_93-x_Ta_x_Al_7_ (x = 3, 5, 7) MPEAs. Low-angle grain boundaries (2–15°) were colored red, and high-angle grain boundaries (>15°) were colored black.

Subsequent TEM analysis (discussed in the following section) revealed that the Ta-rich regions consisted of a lamellar interlaced structure of a Ta-rich hexagonal phase and a Ta-depleted FCC phase, with individual layer widths of approximately ~200 nm. The fine nature of this structure makes it challenging to analyze using EBSD. Moreover, given the alloy’s relatively large grain size, all EBSD measurements were conducted with a 1.5 μm scanning step size to mainly characterize grain morphology and determine the average grain size of the as-cast alloy. Therefore, EBSD failed to detect the hexagonal phase, with all three alloys exhibiting a single FCC phase. The detailed microstructure and elemental distribution within Ta-rich regions will be presented in subsequent TEM characterization. To ensure statistical accuracy in grain-size measurement, EBSD analysis was conducted at lower magnification for the Ta3 alloy, which had the largest grain size, while a higher magnification was used for the Ta5 and Ta7 MPEAs. As evidenced by the inverse pole figure (IPF) maps in Figure 5(a1–c1): As expected, the as-cast alloys exhibited randomly oriented grains without a pronounced texture. Further analysis revealed a progressive refinement in grain size with increasing Ta content, yielding measured grain sizes of 190.7 ± 70.4 μm for Ta3, 110.2 ± 38.5 μm for Ta5, and 51.5 ± 21.9 μm for Ta7. In the grain boundary maps, boundaries with misorientation angles between 2 and 15° were defined as low-angle grain boundaries (LAGBs) and were colored red. Boundaries with misorientation angles greater than 15° were defined as high-angle grain boundaries (HAGBs) and were colored black. The results indicated that a higher proportion of LAGBs was revealed in MPEA with higher Ta content.

**Figure 6:**
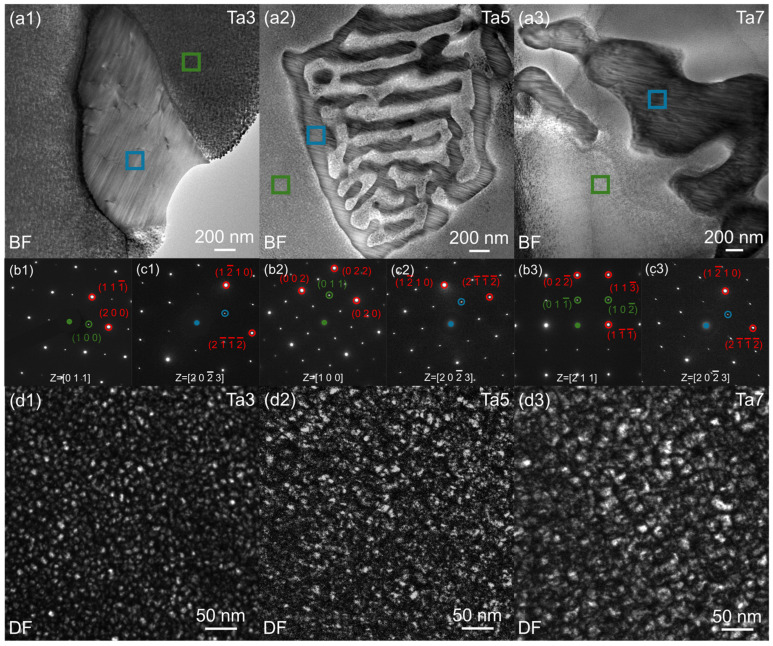
(**a1**–**a3**) TEM BF micrographs of Ta3, Ta5, and Ta7 MPEAs, where the green boxes and blue boxes indicated Ta-depleted matrix regions and Ta-rich secondary phases, respectively; (**b1**–**b3**) SAED patterns corresponding to the matrix regions marked by green boxes; (**c1**–**c3**) SAED patterns from the Ta-rich secondary phase indicated by the blue boxes; (**d1**–**d3**) DF images obtained using the superlattice spots marked by the small green circles in (**b1**–**b3**).

Selected area electron diffraction (SAED) results acquired from the matrix (Figure 6(b1–b3)) further confirmed that the matrix of all alloys exhibited an FCC diffraction pattern. The diffraction patterns (Figure 6(b1–b3)) showed superlattice diffraction spots indicated by the green circles. This suggests that the matrix was not a single-phase FCC solid solution but likely contained L1_2_-type ordered precipitates. These precipitates were attributed to nanoscale ordering involving Ni and Ta, a formation strongly supported by the chemical composition of the matrix in the present alloys (Table 2), which included sufficient Ni (22.2–28.5 at.%) and Ta (3.8–8.1 at.%) contents to facilitate such ordering. Such ordered structures have been previously reported in FeCoNiTaAl alloy systems and described as coherent with the matrix [47,48,49,50]. The SAED patterns obtained from the Ta-rich regions marked by the blue squares (Figure 6(c1–c3)) were consistent with diffraction along the [$20\overset{-}{2}3$] zone axis of the Co_2_Ta-type hexagonal structure. In combination with the XRD results, this conclusively identified the Ta-rich phases as hexagonal. The dark-field images taken from the superlattice spots of the matrix (Figure 6(d1–d3)) revealed distinct L1_2_ ordered phases. Statistical analysis indicated that the volume fractions of the L1_2_ ordered phase in the Ta3, Ta5, and Ta7 alloys were 11.47%, 10.44%, and 10.10%, respectively, with corresponding average sizes of 7.3 ± 2.7 nm, 8.7 ± 4.0 nm, and 10.2 ± 5.7 nm. Regarding the Co_2_Ta-type Laves phase, the appearance of superlattice diffraction spots marked by green circles in the SAED pattern indicated ordering within this phase [60]. The lattice parameters of the matrix in Ta3, Ta5, and Ta7 were a = 0.354 nm, 0.362 nm, and 0.354 nm, respectively. The lattice parameters of the Ta-rich secondary phase were a = 0.460 nm, 0.477 nm, and 0.476 nm, with c = 0.715 nm, 0.740 nm, and 0.731 nm, respectively.

STEM point analysis results (see Table 2) clearly delineated the compositional contrast between the secondary phase and the matrix in the Ta3, Ta5, and Ta7 alloys. The secondary phase was not solely composed of Co and Ta. It also contained significant amounts of Fe, with a Co/Ta ratio ranging from 1.4 to 2.0. In summary, the secondary phases exhibited a Co_2_Ta-type Laves crystal structure rather than strict binary stoichiometry [57,58,59]. In contrast, the FCC matrix exhibited a notably lower Ta content, while the concentrations of Fe, Co, and Ni were comparable to or slightly higher than those in the secondary phase.

**Figure 7:**
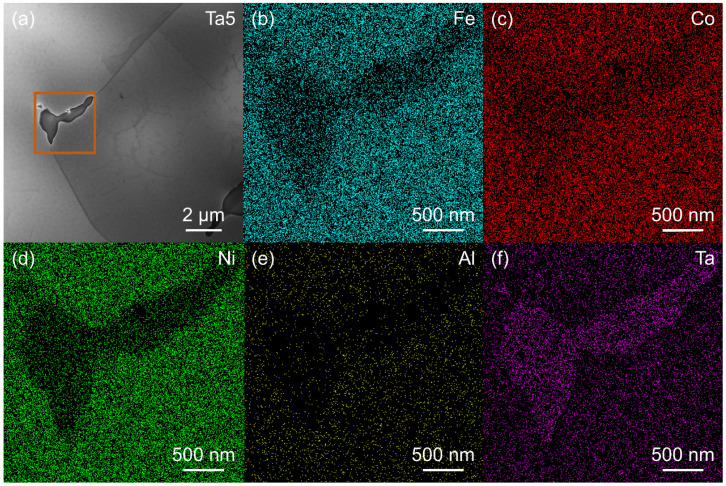
(**a**) STEM BF micrograph of (Fe_7_Co_6_Ni_6_)_90_Ta_3_Al_7_ alloy; (**b**–**f**) EDS elemental maps corresponding to the boxed region in (**a**).

Figure 7a shows a STEM bright-field (BF) micrograph of the Ta3 alloy, where secondary phases precipitated along the grain boundaries, without the lamellar structures observed in Ta5 and Ta7. Figure 7b–f present elemental mapping results for the region marked by the orange square in Figure 6(a1–a3), revealing the distributions of Fe, Co, Ni, Al, and Ta. Consistent with the EPMA-EDS results, the secondary phases were enriched in Ta.

**Figure 8:**
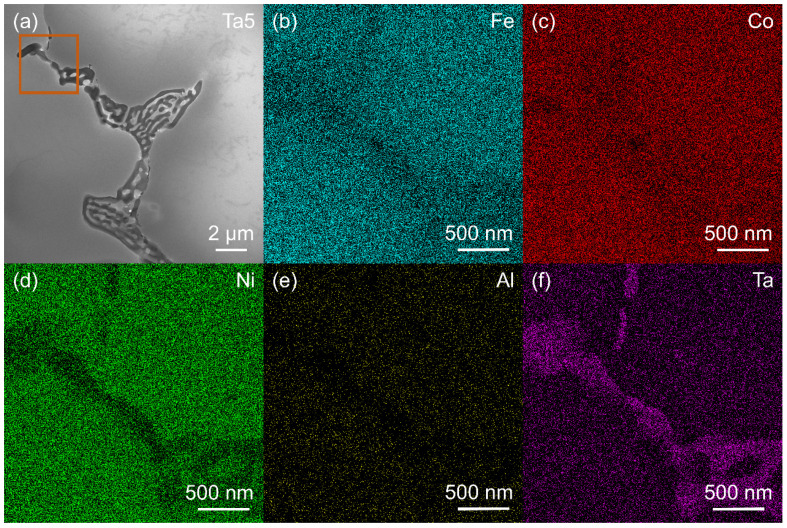
(**a**) STEM BF micrograph of the (Fe_7_Co_6_Ni_6_)_88_Ta_5_Al_7_ alloy; (**b**–**f**) EDS elemental maps corresponding to the boxed region in (**a**).

Figure 8a revealed the lamellar structures along the grain boundaries in the Ta5 alloy. Elemental map results provided in Figure 8b–f revealed that these lamellar structures were composed of Ta-rich secondary phases (Figure 8f) and Ta-depleted regions with compositional characteristics similar to the matrix. Combined with XRD results confirming that the alloy comprised solely an FCC matrix and a Laves phase, these Ta-depleted regions were identified as the FCC phase.

**Figure 9:**
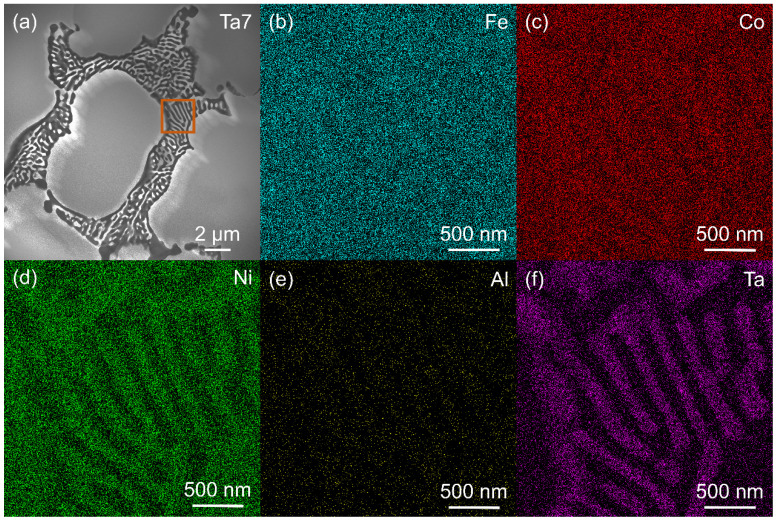
(**a**) STEM BF micrograph of the (Fe_7_Co_6_Ni_6_)_86_Ta_7_Al_7_ alloy; (**b**–**f**) EDS elemental maps corresponding to the marked region in (**a**).

Figure 9a showed a further increase in the volume fraction of secondary phases in the Ta7 alloy. Elemental mapping results (Figure 9b–f) of the lamellar structure region marked in Ta7 revealed a morphology similar to that observed in Ta5: an interconnected distribution of Ta-rich Co_2_Ta-type secondary phases and a Ta-depleted FCC matrix.

**Figure 10:**
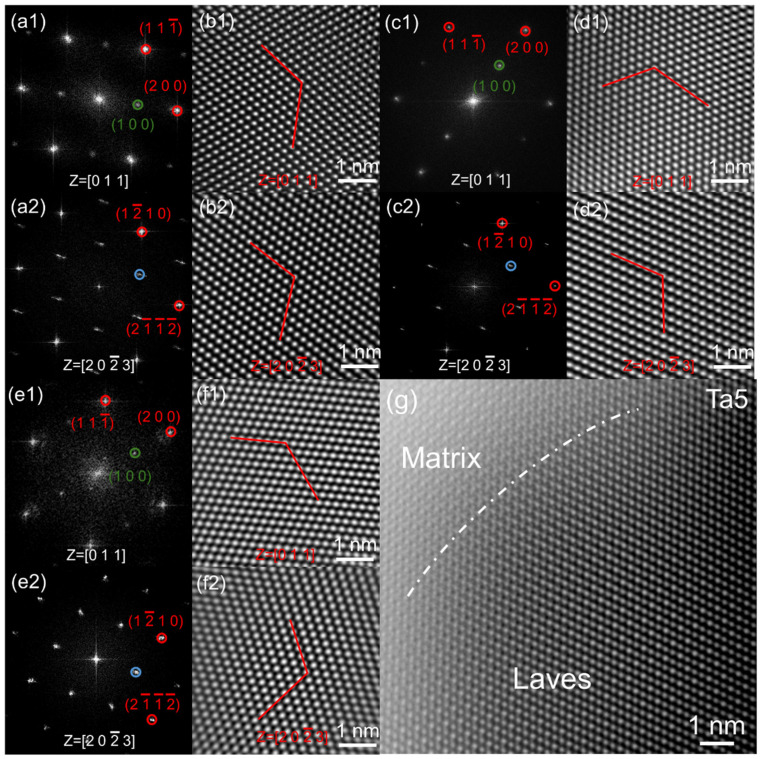
(**a1**,**a2**) FFT patterns acquired from the matrix and the Ta-rich secondary phase of the Ta3 alloy, respectively; (**b1**,**b2**) Corresponding IFFT images of the matrix and secondary phase regions marked in the Ta3 alloy; (**c1**,**c2**) FFT patterns from the matrix and secondary phase of the Ta5 alloy; (**d1**,**d2**) IFFT images of the corresponding regions in the Ta5 alloy; (**e1**,**e2**) FFT patterns from the matrix and secondary phase of the Ta7 alloy; (**f1**,**f2**) IFFT images of the corresponding regions in the Ta7 alloy; (**g**) HR-TEM image of the interface between the matrix and the secondary phase in the Ta5 alloy.

Fast Fourier transform (FFT) patterns were extracted from both the matrix and the Ta-rich secondary phase for the Ta3, Ta5, and Ta7 alloys (Figure 10). The crystal structures derived from these FFT patterns were consistent with the SAED analysis. For the matrix, the FFT patterns (Figure 10(a1–c1)) along the [011] zone axis confirmed an FCC structure. In contrast, the FFT patterns (Figure 10(a2–c2)) from the Ta-rich secondary phase corresponded to a hexagonal structure, indexed along the [$20\overset{-}{2}3$] zone axis, with prominent reflections from the ($1\overset{-}{2}10$) and ($2\overset{-}{1}\overset{-}{1}\overset{-}{2}$) planes. In these IFFT images, the red lines delineated the atomic planes corresponding to the specific reflections selected in the FFT. Figure 10g shows the HR-TEM image of the interface between the matrix and Laves phase in the Ta5 alloy. Across different zone axes, the lattice fringes of the Laves phase and the matrix could not be brought into clear focus simultaneously. In combination with the XRD results and the significant difference in lattice parameters between the two phases observed in the SAED patterns, it can be concluded that the interface between the two phases in the Ta5 alloy was incoherent.

### 3.2. Magnetic and Mechanical Properties

The volume fraction of the secondary phase was quantitatively determined from backscattered electron (BSE) images. First, the area fraction of Ta-rich regions was evaluated in low-magnification BSE micrographs using ImageJ 1.54f software, yielding values of 1.37%, 11.34%, and 21.06% for the Ta3, Ta5, and Ta7 alloys, respectively. To account for the multiphase nature within Ta-rich regions for Ta5 and Ta7, higher-magnification TEM analysis was adopted to distinguish the Laves phase from the surrounding matrix. The area proportion of the Laves phase within the Ta-rich regions was found to be 70.7% for Ta5 and 75.8% for Ta7. By combining these two sets of measurements, the final volume fractions of the Laves phase were calculated as 1.37%, 8.23%, and 15.96% for the Ta3, Ta5, and Ta7 alloys, respectively.

**Figure 11:**
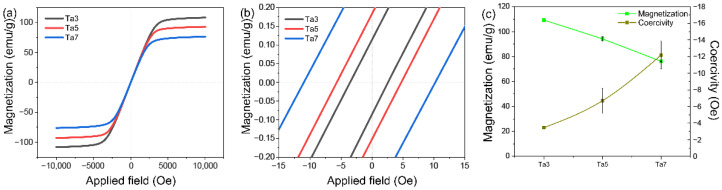
(**a**) Room-temperature magnetization measurements of the (Fe_7_Co_6_Ni_6_)_93-x_Ta_x_Al_7_(x = 3, 5, 7) alloys; (**b**) a partial enlarged view of the hysteresis loops near the origin; (**c**) magnetization and coercivity vs. Ta content of Ta3, Ta5, and Ta7 alloys.

Figure 11a shows the room temperature saturation magnetization of Ta3, Ta5, and Ta7 alloys, while Figure 11b presents a magnified view of the region near the origin of (a). The intersection of the hysteresis loop with the negative *x*-axis defines the coercivity of an alloy; relevant parameters of MPEAs are summarized in Table 3. As shown in Figure 11 and Table 3, the M_s_ of MPEAs decreased with increasing Ta content, from 109.28 emu/g to 76.19 emu/g. Since Ta is non-ferromagnetic and cannot be magnetized, the M_s_ of MPEAs exhibited a positive correlation with the fraction of ferromagnetic elements (Fe, Co, Ni) (Figure 11c). This reduction correlated linearly with the observed decrease in M_s_, indicating a strong positive dependence of M_s_ on the ferromagnetic element content. Regarding microstructural effects, the grain size in this alloy system decreased significantly from 190.7 μm to 51.5 μm. It should be noted that while grain refinement increased the volume fraction of grain boundaries, which could slightly reduce M_s_. However, this effect was limited. Given the large grain size, the variation in M_s_ arising solely from grain-boundary area was typically small [41,61]. Therefore, the observed change in M_s_ was predominantly governed by compositional dilution.

Regarding coercivity, as the Ta content increased from 3 at.% to 5 at.%, the coercivity rose from 3.45 Oe to 6.69 Oe. When the Ta content reached 7 at.%, the coercivity increased sharply to 12.18 Oe. Coercivity is widely recognized to originate from the pinning of magnetic domain walls by various microstructural features. The primary factors responsible for the increased coercivity in this study were the significant increase in the volume fraction of the Laves phase (from 1.37% to 15.96%) and the pronounced grain refinement (grain size decreasing from 190.7 μm to 110 μm, then to 51.5 μm). The Laves phases, particularly at grain boundaries, acted as strong pinning sites for domain walls. Concurrently, the increased density of high-angle grain boundaries due to refinement further impeded domain-wall motion. The nanoscale L1_2_ ordered precipitates (volume fractions of ~11.5–10.1%, sizes of ~7–10 nm) also introduced additional interfaces; however, their coherent nature with the matrix and nanoscale size resulted in a minor contribution to the overall coercivity compared to the Laves phases and grain boundaries [54]. Therefore, the observed trend in coercivity was primarily governed by the evolution of the Laves phase and the matrix grain size. Specifically, the combined effects of increased volume fraction and size of Laves phases, as well as grain refinement with increasing Ta content, collectively enhanced domain-wall pinning, thereby increasing coercivity, which was particularly evident in the Ta7 alloy. Yet, its value remained well below the 1000 A/m (~12.6 Oe) benchmark for soft magnetic materials.

**Figure 12:**
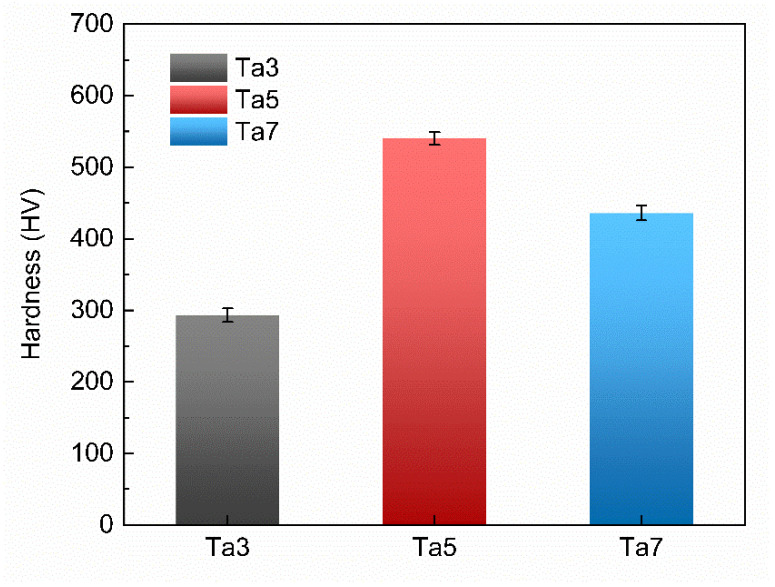
Vickers hardness values of the (Fe_7_Co_6_Ni_6_)_93-x_Ta_x_Al_7_ (x = 3, 5, 7) MPEAs.

Figure 12 demonstrates the influence of Ta content on alloy hardness. The Ta3 alloy exhibited the lowest hardness (293.2 ± 9.5 HV), which can be attributed to its predominant softer FCC/L1_2_ phase and the lowest volume fraction of the Laves phase among the three alloys. As the Ta content increased, the higher proportion of the hard Laves phase contributed to enhanced alloy hardness. It is noteworthy that the hardness of the Ta7 alloy (435.8 ± 10.2 HV) decreased relative to that of Ta5 (540.1 ± 8.8 HV). This was likely due to the greater brittleness of Ta7 compared to Ta3 and Ta5, which led to surface fracture under the pressure of the hardness indenter. The resulting reduction in the actual indentation area consequently led to a lower measured hardness value.

**Figure 13:**
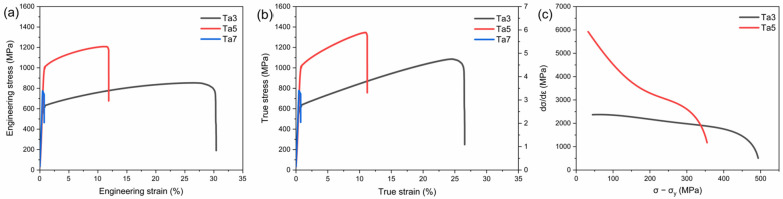
(**a**) Engineering stress–strain curves of the (Fe_7_Co_6_Ni_6_)_93-x_Ta_x_Al_7_ (x = 3, 5, 7) alloys under tensile testing at a strain rate of 1 × 10^−3^ s^−1^; (**b**) true stress–strain curves from (**a**); (**c**) Kocks–Mecking plots (work hardening rate vs. net flow stress) for the Ta3 and Ta5 alloys.

Figure 13a shows the tensile engineering stress–strain curves of the MPEAs. The Ta3 alloy exhibited the lowest yield strength (595 MPa) but the highest ductility, with an average tensile elongation (TE) of 29.3% (Table 4). This behavior was consistent with its microstructure, which consisted of a coarse-grained (190.7 μm), ductile FCC matrix strengthened only by nanoscale L1_2_ precipitates (11.47 vol.%, 7.3 ± 2.7 nm) and a minimal amount of Laves phase (1.37 vol.%). In contrast, the Ta5 alloy achieved an optimal balance among the three alloys, demonstrating the highest yield and ultimate tensile strengths (993 MPa and 1210 MPa, respectively) while retaining an acceptable elongation of 10.3%. Its high strength originated from multiple strengthening mechanisms: (1) precipitation strengthening from the substantially increased volume fraction of hard Laves phases (8.23 vol.%); (2) precipitation strengthening from a high density of coherent, nanoscale L1_2_ precipitates (10.44 vol.%, 8.7 ± 4.0 nm) within the FCC matrix; and (3) grain boundary strengthening due to grain refinement (110.2 μm in Ta5 vs. 190.7 μm in Ta3). The Ta7 alloy underwent brittle fracture with negligible plastic elongation (0.6%). This severe embrittlement was directly attributable to the high volume fraction of Laves phase (15.96 vol.%), which drastically reduced intergranular cohesion and provided easy routes for crack propagation.

The work-hardening behavior, analyzed using Kocks–Mecking (K–M) plots (Figure 13c), provides further insight into the deformation mechanisms. As shown in Figure 13b, the Ta5 alloy exhibited a higher initial work hardening rate than Ta3. This was attributed to its finer grain size (110.2 μm vs. 190.7 μm)—a dominant factor influencing the initial work hardening rate of alloys [62]—and, more importantly, to the high density of Laves phases. These dispersed obstacles effectively impeded dislocation motion, leading to rapid dislocation storage and a higher hardening rate at the onset of plastic deformation. The K–M plots (Figure 13c), which depict the work hardening rate (θ) as a function of flow stress (σ), revealed that while Ta5 started with a higher θ, its value decreases more rapidly with increasing stress compared to Ta3. This accelerated decline indicated a transition to a regime in which the rate of dislocation storage was increasingly balanced by dislocation rearrangement and annihilation. The hard Laves phases and the refined grain structure in Ta5 accelerated the transition from dislocation accumulation to the build-up of a stable, high-density dislocation network. The development of such a saturated dislocation structure reduced the efficiency of subsequent dislocation storage, as evidenced by a more rapid decline in the work-hardening rate [63]. The convergence of the θ values for Ta3 and Ta5 at higher stresses indicated that dislocation-dislocation interactions gradually became the dominant hardening mechanism in the later stages of deformation, overshadowing the initial effects of grain boundaries and precipitates [64,65].

To establish a quantitative correlation between microstructure and strength, the yield strength σ_y_ is decomposed as [27,66,67]:

(1) $$\sigma_{y}=\sigma_{ss}+\sigma_{gb}+\sigma_{pl}+\sigma_{L1_{2}}+\sigma_{Laves}$$

where σ_ss_ = 277 MPa is the solid-solution strengthening [66]; σ_gb_ and σ_pl_ are the grain-boundary and dislocation strengthening, respectively, evaluated using standard Hall-Petch and Taylor relations; σ_L12_ accounts for order strengthening from shearing of coherent L1_2_ nanoprecipitates, calculated according to Ref. [66]; and σ_Laves_ represents the contribution of the incoherent Laves phase.

The calculated strengthening contributions to yield strength were summarized in Table 5. For the Ta3 alloy, its yield strength (595 MPa) mainly originated from solid-solution strengthening (277 MPa) and precipitation strengthening of the L1_2_ phase (~312 MPa), while the contribution from the Laves phase was minor (~11 MPa). The calculated strength (636 MPa) reasonably agreed with the measured yield strength (595 MPa). For the Ta5 alloy, the calculated strengthening from the first four terms (622 MPa) was substantially lower than the experimental yield strength (993 MPa), revealing a large strengthening gap (~371 MPa) that may originate from the Laves phases, which was not easy to estimate by a simple analytical model due to the complex morphology comprising both lamellar and particulate features. For the Ta7 alloy, the calculated strengthening from the first four terms (640 MPa) cannot be directly compared with the experimental result, as the alloy fractured in a brittle manner and did not exhibit macroscopic yielding.

**Figure 14:**
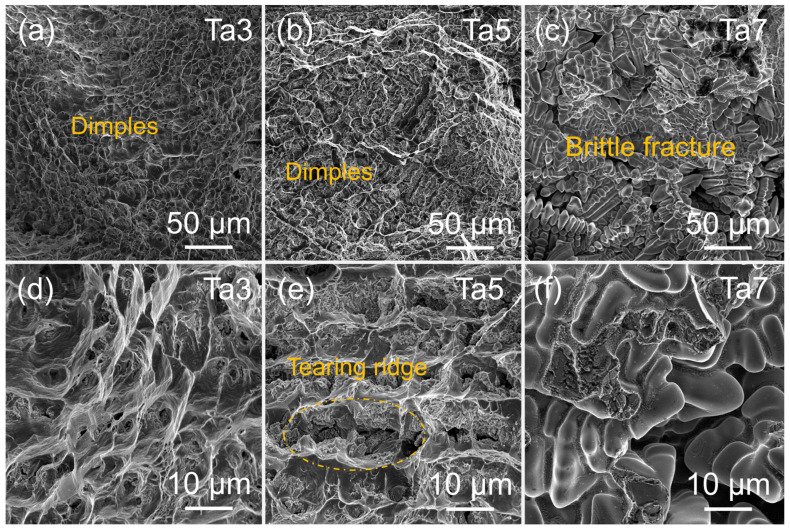
(**a**–**c**) The fracture morphology after tensile testing of MPEAs at lower magnification, (**d**–**f**) the fracture morphology after tensile testing of MPEAs at higher magnification.

Fracture morphologies of the three alloys (Figure 14) were analyzed to elucidate their fracture mechanisms. In the Ta3 alloy, the volume fraction of Laves phase was only 1.37%, appearing as isolated particles, and the alloy exhibited excellent ductility (29.3% elongation) with a fracture surface covered by numerous fine dimples, indicating a typical ductile fracture. When the Ta content increased to 5 at.%, the particulate Laves phase transformed into lamellar structures along grain boundaries, with the volume fraction rising to 8.23%, providing substantial precipitation strengthening (~370 MPa) that elevated the yield strength to 993 MPa while retaining an elongation of 10.3%. Its fracture morphology exhibited a limited number of dimples and tear ridges, reflecting severe localized plastic deformation. However, with Ta content further increased to 7 at.%, the Laves phase volume fraction reached 15.96%, which embrittled the alloy, reducing elongation to only 0.6%. The excessive Laves phase, although enhancing strength, triggered brittle fracture due to its high brittleness, severely compromising ductility [58]. In summary, by tailoring the Ta content, the volume fraction and morphology of Laves phases can be modified to achieve a balance between strength and ductility in FeCoNiAlTa-based alloys.

In brief, as Ta content increased, grain size refined progressively; the Laves phase transformed from isolated particles into a lamellar structure with a marked increase in volume fraction, while the L_12_ phase showed a slight decrease in volume fraction but a minor increase in size. These microstructural changes directly translated into the observed property trends—yield strength was optimized at Ta5, while Ta7 exhibited intergranular brittle fracture due to excessive Laves phase aggregation; M_s_ decreased from ferromagnetic dilution, and Hc increased from enhanced pinning at Laves phases and grain boundaries.

**Figure 15:**
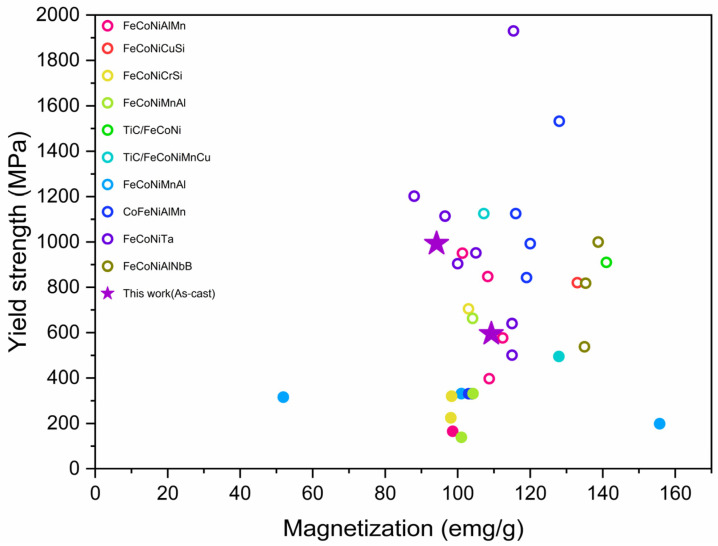
Comparison of yield strength versus M_s_ for the as-cast alloys in this study and other reported soft magnetic MPEAs [41,47,48,49,53,68,69,70,71,72,73,74], with open and solid circles denoting thermomechanically processed and as-cast alloys, respectively.

In soft magnetic materials, M_s_, coercivity, and yield strength are key parameters governing performance. Figure 15 compares the yield strength and M_s_ of the alloys in this work with those of other soft-magnetic FeCoNi-based MPEAs, specifically those with a coercivity below 1000 A/m. The results demonstrated an outstanding combination of properties in as-cast Ta3 and Ta5 alloys compared to other as-cast MPEAs. Notably, the superior performance of the Ta3 and Ta5 alloys is comparable to that of some thermomechanically processed FeCoNi-based MPEAs [41,47,48,49,53,68,69,70,71,72,73,74].

To sum up, this work established the critical role of Ta in tailoring the microstructure and properties of as-cast (Fe_7_Co_6_Ni_6_)_93-x_Ta_x_Al_7_ MPEAs, with SEM and TEM providing direct evidence for the core structure–property correlations. To gain deeper mechanistic insights, future work can employ advanced nanoscale characterization: atomic force microscopy (AFM) for mapping the nanoscale phase distribution and mechanical heterogeneity within the alloy, piezoresponse force microscopy (PFM) for probing potential local magnetoelectric or polar responses linked to chemical ordering, and secondary ion mass spectrometry (SIMS) for tracking the precise depth distribution and segregation behavior of alloying elements like Ta at interfaces and grain boundaries [75,76]. These techniques will collectively elucidate how nanoscale chemical and structural heterogeneities govern the macroscopic magnetic and mechanical response of the alloy. In addition, rolling with heat treatment could further reduce grain size and potentially alter the morphology and distribution of the Laves and L1_2_ phases, thereby affecting the balance between strength, ductility, and magnetic properties. Regarding magnetic properties, heat treatment may reduce coercivity by reducing the size of Laves and L1_2_ phases, while the saturation magnetization (M_s_) is expected to remain largely unchanged, as it is primarily governed by composition.

## 4. Conclusions

1. The Ta5 alloy (x = 5) exhibited the best performance in the (Fe_7_Co_6_Ni_6_)_93-x_Ta_x_Al_7_ system, achieving a yield strength of 993 MPa, an elongation of 10%, an M_s_ of 94.16 emu/g, and a coercivity of 6.69 Oe, which represented an excellent balance of strength, ductility, and soft magnetic properties compared to other as-cast MPEAs.
2. Magnetic properties were a direct result of both compositional and microstructural factors. The increase in Ta content diluted the ferromagnetic elements, reducing M_s_. Concurrently, the presence of Laves phases along grain boundaries and the refinement of grains themselves enhanced domain-wall pinning, thereby raising coercivity. Notably, for the Ta5 alloy, this increase remained modest.
3. Mechanical properties were dictated by microstructural evolution. A moderate Ta content (x = 5) enhanced strength via synergistic strengthening from the coherent nanosized L1_2_ phase, Co_2_Ta-type lamellar Laves phases, and grain refinement. High Ta content (x = 7) led to excessive Laves phases at grain boundaries, resulting in intergranular fracture and loss of ductility.

## Figures and Tables

**Table 1:** The atomic ratio, mass ratio, and theoretical density of MPEAs.

Alloy	Fe (wt.%)	Co (wt.%)	Ni (wt.%)	Ta (wt.%)	Al (wt.%)	ρ (g/cm^3^)
(Fe_7_Co_6_Ni_6_)_90_Ta_3_Al_7_	31.25	28.26	28.15	9.16	3.19	8.29
(Fe_7_Co_6_Ni_6_)_88_Ta_5_Al_7_	29.33	26.53	26.42	14.66	3.06	8.54
(Fe_7_Co_6_Ni_6_)_86_Ta_7_Al_7_	27.56	24.93	24.83	19.73	2.94	8.78

**Table 2:** Point analysis results for the matrix and secondary phases in Ta3, Ta5, and Ta7 MPEAs.

		Fe (at.%)	Co (at.%)	Ni (at.%)	Ta (at.%)	Co/Ta
Ta3	Laves	25.6	31.2	19.7	22.0	1.4
	Matrix	30.9	28.7	28.4	7.6	
Ta5	Laves	29.0	36.8	14.6	18.7	2.0
	Matrix	39.9	33.7	22.2	3.8	
Ta7	Laves	26.9	30.1	18.8	21.9	1.4
	Matrix	29.5	29.8	28.5	8.1	

**Table 3:** Magnetic properties of Ta3, Ta5, and Ta7 alloys obtained from Figure 11.

Alloy	M_s_ (emu/g)	H_c_ (Oe)	H_c_ (A/m)
Ta3	109.28 ± 1.50	3.45 ± 0.12	274.5 ± 9.5
Ta5	94.16 ± 1.81	6.69 ± 1.53	532.4 ± 121.8
Ta7	76.19 ± 0.13	12.18 ± 1.68	969.3 ± 133.7

**Table 4:** Yield strength, ultimate tensile strength, and elongation of the (Fe_7_Co_6_Ni_6_)_93-x_Ta_x_Al_7_ (x = 3, 5, 7) alloys. The yield strength of Ta alloy cannot be determined due to the completely brittle fracture.

Alloy	σ_s_ (MPa)	σ_UTS_ (MPa)	TE (%)
Ta3	595 ± 26	849 ± 38	29.3 ± 3.8
Ta5	993 ± 12	1210 ± 83	10.3 ± 3.1
Ta7	--	799 ± 153	0.6 ± 0.2

**Table 5:** Calculated strengthening contributions (MPa) for the (Fe_7_Co_6_Ni_6_)_93-x_Ta_x_Al_7_ (x = 3, 5, 7) MPEAs.

Alloy	σ_ss_ (MPa)	σ_gb_ (MPa)	σ_pl_ (MPa)	σ_L12_ (MPa)
Ta3	277	33.5	2.2	311.9
Ta5	277	44.0	3.4	297.6
Ta7	277	64.4	5.7	292.8

## Data Availability

The original contributions presented in this study are included in the article/Supplementary Materials. Further inquiries can be directed to the corresponding author.

## Supplementary Materials

The following supporting information can be downloaded at: https://www.mdpi.com/article/10.3390/ma19122509/s1. Supplementary: Calculation of strengthening contributions.
