# Supplementary material for: Tailoring Mechanical and Soft Magnetic Properties in (Fe7Co6Ni6)93-xTaxAl7 Multi-Principal Element Alloys: The Role of Ta Addition
Source: Materials (Basel). 2026 Jun 10;19(12):2509. doi: 10.3390/ma19122509 (PMC13303066; doi:10.3390/ma19122509)
Supplement: Supplementary file 1 [file materials-19-02509-s001.zip › materials-4054082-supplementary.pdf]

Supplementary Material

To further elucidate the mechanical properties of the alloys, the particle size and inter-particle spacing of the secondary phase in the Ta3 alloy were statistically measured from Fig. 2b, yielding an average particle size of 1.63 ± 1.02 μm and an average spacing of 7.27 ± 6.19 μm. The average interlamellar spacing of the secondary phase in the Ta5 and Ta7 alloys was measured from the Figs. 7a and 8a, resulting in values of 266.3 ± 40.7 nm and 318.3 ± 64.6 nm, respectively.

To establish a quantitative correlation between the geometric parameters of the secondary phase and the macroscopic mechanical performance, the approximate yield strength of the alloys can be expressed as [27,66,67]:

$$\sigma_y = \sigma_{ss} + \sigma_{gb} + \sigma_{pl} + \sigma_{L1_2} + \sigma_{Laves} \quad (S1)$$

Here, σ_{ss} denotes the solid-solution strength of the matrix and was taken as 277 MPa [66]. The term σ_{gb} denotes the yield strength contribution from grain boundary (Hall-Petch) strengthening. σ_{pl} corresponds to the work hardening associated with the effective dislocation density, σ_{L12} calculates the strength provided by the shearing of coherent $L1_2$ precipitates by dislocations, and σ_{Laves} represents the strengthening contribution from incoherent Laves phases.

The contribution of grain boundary strengthening is calculated using the Hall-Petch relationship:

$$\sigma_{gb} = k_{H-P} d^{-1/2} \quad (S2)$$

Where k_{H-P} = 462 MPa μm^(1/2) is a typical value for FCC-based alloys within the FeCoNiAlTa system. With increasing Ta content, the grain size is significantly refined from 190.7 μm for Ta3 to 110.2 μm for Ta5, and further to 51.5 μm for Ta7. Consequently, the grain boundary strengthening contribution increases from 33.5 MPa for Ta3 to 44.0 MPa for Ta5, and finally to 64.4 MPa for Ta7.

The dislocation strengthening is estimated using the Taylor formula:

$$\sigma_{pl} = M \alpha G b \rho^{-\frac{1}{2}} \quad (S3)$$

Where M = 3.06 is the Taylor factor (a typical value for FCC crystals), α = 0.2 is an empirical constant, G = 75 GPa is the shear modulus, and b = 0.2556 nm is the magnitude of the Burgers vector. The dislocation density increases with Ta content from 3.60×10^{10} m^{-2} for Ta3 to 8.28×10^{10} m^{-2} for Ta5, and reaches 2.32×10^{11} m^{-2} for Ta7, leading to a progressive increase in the dislocation

strengthening contribution from 2.2 MPa for Ta3, to 3.4 MPa for Ta5, and to 5.7 MPa for Ta7.

The strengthening due to dislocation shearing through coherent $L1_2$ nanoprecipitates can be estimated using the following expression[66]:

$$\sigma_{L1_2} = 0.81\, M \left(\frac{\gamma_{APB}}{2b}\right) \left(\frac{3\pi f}{8}\right)^{1/2} \tag{S4}$$

Here, b is the Burgers vector magnitude calculated from the lattice parameter of the matrix (b $= a/\sqrt{2} = 0.2556$ nm), $\gamma_{APB} = 175$ mJ·m^{-2} is the anti-phase boundary energy for $L1_2$ precipitates in the FeCoNiAlTa system, and f is the volume fraction of the $L1_2$-ordered phase, obtained from TEM dark-field images (Figs. 9d1-d3) with values of 0.1147, 0.1044, and 0.1010 for Ta3, Ta5, and Ta7, respectively. Using these parameters, the calculated $L1_2$ strengthening contributions are 311.9 MPa for Ta3, 297.6 MPa for Ta5, and 292.8 MPa for Ta7.

The Laves phase in the three alloys exhibited distinct morphologies: isolated particles in Ta3, both particles and lamellar structures in Ta5 and Ta7, with a substantially higher volume fraction in the latter. This morphological complexity, together with the brittle fracture of Ta7, makes it impractical to quantify the strengthening contribution of the Laves phase using a unified model.
